# The use of glycoinformatics in glycochemistry

**DOI:** 10.3762/bjoc.8.104

**Published:** 2012-06-21

**Authors:** Thomas Lütteke

**Affiliations:** 1 Justus-Liebig-University Gießen, Institute of Veterinary Physiology and Biochemistry, Frankfurter Str. 100, 35392 Gießen, Germany

**Keywords:** carbohydrates, databases, glycomics, software

## Abstract

Glycoinformatics is a small but growing branch of bioinformatics and chemoinformatics. Various resources are now available that can be of use to glycobiologists, but also to chemists who work on the synthesis or analysis of carbohydrates. This article gives an overview of existing glyco-specific databases and tools, with a focus on their application to glycochemistry: Databases can provide information on candidate glycan structures for synthesis, or on glyco-enzymes that can be used to synthesize carbohydrates. Statistical analyses of glycan databases help to plan glycan synthesis experiments. 3D-Structural data of protein–carbohydrate complexes are used in targeted drug design, and tools to support glycan structure analysis aid with quality control. Specific problems of glycoinformatics compared to bioinformatics for genomics or proteomics, especially concerning integration and long-term maintenance of the existing glycan databases, are also discussed.

## Introduction

Carbohydrates, often referred to as glycans, differ from other biopolymers such as proteins or nucleic acids in various ways. The number of different monosaccharides that are present in naturally occurring glycans is significantly higher than the number of proteogenic amino acids, or of nucleotides that form DNA or RNA strands [1–2]. Furthermore, the monosaccharides can be linked to each other in several ways, including the possibility to form branched structures. Another important difference between glycans, on the one hand, and proteins and nucleic acids, on the other hand, is visible in their biosynthesis: DNA, RNA and proteins are synthesized by copying, transcription or translation, respectively, of nucleic acids, whereas carbohydrates are built in a non-template-driven approach by the sequential action of various glycosyltransferases (GT) that add monosaccharides to an existing glycan chain, and by glycoside hydrolases (GH) that remove specific monosaccharides [3]. For this reason there is no technique available to amplify carbohydrates comparable to Polymerase Chain Reaction (PCR) or protein expression systems. Instead, carbohydrates have to be analyzed in physiological amounts. If specific and well-defined glycans are required for experiments such as glycan arrays [4], they have to be synthesized chemically [5].

The special features of carbohydrates not only pose problems for their wet-lab analysis but also for computational approaches that deal with carbohydrates. Classical bioinformatics algorithms are developed for linear gene or protein sequences, and thus cannot be applied to branched carbohydrates. Instead, new algorithms that deal with the branching as well as with other special features of carbohydrates, such as microheterogeneity, have to be developed [6–7]. Furthermore, there are much less primary data on carbohydrates available than, e.g., on proteins, to test or train the algorithms [8]. For these reasons, glycoinformatics as a research area at the intersection of bioinformatics and chemoinformatics has been considered to be lagging behind its sister fields, such as bioinformatics for genomics or proteomics, for a long time. By now, however, glycoinformatics is coming of age and offers a variety of databases and applications that are of use to glycoscientists. Many new resources are still being developed, and efforts for a better integration of existing resources have also been started. Formats and protocols for data exchange have been specified [9–10]. Recently, the MIRAGE (Minimum Information Related to A Glycomics Experiment) consortium was founded to define checklists for the standardization of experimental glycomics data and meta information [11]. However, there is still no long-term repository of glycan structures available.

Being part of the Thematic Series “Synthesis in the glycosciences II”, this overview mainly focuses on those resources that are relevant to glycan synthesis. For a more general overview of glycoinformatics resources and the development of the field over time, the reader is referred to references [12–17].

## Review

### Carbohydrate databases

#### Glycan structure databases

Various databases that collect information on carbohydrates are now available (Table 1) and new resources are still being developed. The individual databases differ in the kind of data that are stored, the number and topicality of entries, the search interfaces, and the way the data are presented to the user. They are of use to glycochemists in several ways. First of all, they provide literature references on specific carbohydrate structures, which are often difficult to find via keyword searches in general literature databases such as PubMed. However, keeping databases up to date with bibliographic references is a time-consuming task that cannot be performed automatically by computer programs because the glycan structures are often encoded graphically within the publication figures. And even when information on glycan chains is given in the text, the notation is often complex, difficult to parse, and may contain ambiguities. Therefore, database users should keep in mind that if a database does not list any reference that, e.g., deals with the synthesis of a specific glycan structure, it does not mean that there is no such reference available: it just might not have been included into the database yet. Aside from providing literature references, carbohydrate databases can also serve glycochemists as a source of information on structures that are potential targets for synthesis. For this purpose resources that feature data such as the biological source, or diseases related to a glycan structure, can be of special interest.

**Table 1:** Carbohydrate structure databases.

Name, Ref	Main content, comments	URL	Status^a^

BCSDB (Bacterial Carbohydrate Structure Database) [18]	Glycan sequences, taxonomy, bibliography, NMR data	http://csdb.glycoscience.ru/bacterial/	D/M
CCSDB//CarbBank [19–20]	Glycan sequences, taxonomy, bibliography	–	S
CFG Glycan Structure DB [21]	Glycan sequences, taxonomy, bibliography, glycan array data	http://www.functionalglycomics.org/glycomics/molecule/jsp/carbohydrate/carbMoleculeHome.jsp	S
ECODAB (*Escherichia coli* O-antigen Database) [22–23]	Glycan sequences, NMR data, glycosyltranferases (*E. coli* glycans only)	http://www.casper.organ.su.se/ECODAB/	D/M
EUROCarbDB [24]	Glycan sequences, taxonomy, bibliography, MS data	http://www.ebi.ac.uk/eurocarb/	S
Glycobase (Dublin) [25]	Glycan sequences, taxonomy, HPLC data, MS data, bibliography	http://glycobase.nibrt.ie (registration required)	D/M
Glycobase (Lille)	Glycan sequences, taxonomy, NMR data	http://glycobase.univ-lille1.fr/base/	?
GlycoconjugateDB [26]	Glycan sequences, PDB references	http://www.glycostructures.jp	?
GlycoMapsDB [27]	Computed conformational maps	http://www.glycosciences.de/modeling/glycomapsdb/	P
GlycoNavi (JCGGDB)	Chemical reactions of carbohydrate molecules	http://ws.glyconavi.org	D/M
GlycomeDB [28–30]	Glycan sequences and taxonomic data extracted from other databases	http://www.glycome-db.org	D/M
GlycoPOD (JCGGDB GlycoProtocols Online Database)	Protocols for synthesis and analysis of glycan structures	http://jcggdb.jp/GlycoPOD/protocolListShow.action	D/M
Glycosciences.DB [31]	Glycan sequences, taxonomy, bibliography, 3D structure models, NMR data, PDB references	http://www.glycosciences.de/database/	D/M
GlycosideDB (JCGGDB)	Chemical structures of glycoconjugates, aglycones	http://jcggdb.jp/search/GlycosideDB.cgi	D/M
GlycoSuiteDB [32–33]	Glycan sequences, taxonomy, bibliography, disease, protein glycosylation sites	http://glycosuitedb.expasy.org	D/M
JCGGDB [34]	Collection of several Japanese glyco-related databases	http://jcggdb.jp/index_en.html	D/M
KEGG GLYCAN [35]	Glycan sequences, bibliography, cross-references to other KEGG resources	http://www.genome.jp/kegg/glycan/	?
MonosaccharideDB	Monosaccharide notation and properties	http://www.monosaccharidedb.org	D/M
SugaBase [36]	Glycan sequences, taxonomy, NMR data	–	S
UniCarb-DB [37]	Glycan sequences, taxonomy, LC–MS data	http://www.unicarb-db.com	D/M

^a^D/M: Database is further developed and/or maintained; P: Development/maintenance is paused, but planned to be continued; S: Development/maintenance is stopped (or, in the case of CFG resources, will be stopped soon because funding will discontinue); ?: Status unknown.

The first approach used to create a comprehensive collection of carbohydrate data that had been published in scientific literature, was the Complex Carbohydrate Structure Database (CCSDB) [19], which is often referred to by the name of its querying software, CarbBank [20]. Each CarbBank entry contains a glycan structure together with a bibliographic reference, and further information such as taxonomic data of the biological source, experimental methods, or related diseases are also present. When funding stopped in the mid-1990s, however, CarbBank was no longer updated and CCSDB no longer maintained. Nevertheless, its content formed the basis of several other databases that were subsequently developed. Depending on their focus, other resources have incorporated all or part of the CarbBank data, and have added further information. The Bacterial Carbohydrate Structure Database (BCSDB), for example, has incorporated ca. 4000 CarbBank entries, the structures of which are of bacterial origin, and added ca. 5000 records referring to articles that were published after the maintenance of CarbBank had stopped. BCSDB covers more than 90% of the literature in the scope of bacterial carbohydrates [18]. Data that are listed in the entries include bibliographic data, taxonomic information on the biological source, and primary data of nuclear magnetic resonance (NMR) experiments that had been performed to elucidate the structures. Other sources of NMR data are SugaBase [36], which similar to CarbBank is no longer maintained, GlycoBase (Lille), *Escherichia coli* O-antigen Database (ECODAB) [22], and Glycosciences.DB [31], the database of the Glycosciences.de web portal. Glycosciences.DB, formerly known as SweetDB [38], started to make CarbBank entries available over the internet and to provide 3D structural models of the glycan structures. These models are calculated by the Sweet-2 software [39]. NMR data were incorporated from SugaBase or manually entered from the literature. Glycosciences.DB also contains information on carbohydrate 3D structures that are available in the Protein Data Bank (PDB, [40]). Extraction and validation of carbohydrate data from PDB entries is automated to a large extent and therefore requires only minimal human interference [41], making the update of these data much less dependent of funding than that of data extracted from the literature. Carbohydrate data from the PDB are also available in the Glycoconjugate Database [26], but updates are less frequent than in Glycosciences.DB, which is updated weekly with new PDB entries.

Other databases that implemented CarbBank data are KEGG GLYCAN [35], EUROCarbDB [24], and the Glycan Structure Database of the Consortium for Functional Glycomics (CFG) [21]. KEGG GLYCAN is part of the Kyoto Encyclopedia of Genes and Genomes (KEGG) and integrates carbohydrate data with a variety of tools and information on other biomolecules. The KEGG portal has a particular focus on biosynthetic pathways. EUROCarbDB was developed to store primary data of mass spectrometry (MS), NMR and high performance liquid chromatography (HPLC) experiments. In addition to data imported from CarbBank, the database contains structures from such experiments (mainly MS data). Detailed information on the biological context in which a structure was found, is also provided. EUROCarbDB is another example of a database that is no longer being developed because the funding has stopped. However, it was developed as an open-source project. Therefore, newly funded projects such as UniCarb-DB [37] or the latest version of GlycoBase (Dublin) [25], which stores HPLC data, are able to make use of the EUROCarbDB source code and, thus, the software does not need to be rewritten. The CFG databases are focused on various aspects of mammalian glycans. Similar to EUROCarbDB, the CFG Glycan Structure Database features CarbBank *N*- and *O*-glycan data as well as entries that have been found in MS experiments performed by CFG members or that have been synthesized by the CFG. The database is complemented with glycans from the GlycoMinds Ltd. seed database. Primary data of MS experiments and glycan array screens are also available via the CFG website. If a glycan structure has been detected to be bound by a glycan-binding protein in a CFG glycan array experiment, links to the corresponding protein pages are provided with the glycan structure entries. A link to a 3D structural model generated by the GLYCAM-Web Biomolecule Builder [42] is also given. GlycoSuiteDB [32–33], which started as a commercial database and was later made publicly accessible, differs from most other carbohydrate databases in that it provides information on proteins to which specific glycans were found to be attached, including details on glycosylation sites.

The Japan Consortium for Glycobiology and Glycotechnology DataBase (JCGGDB) [34] provides a collection of individual databases that are cross-linked with each other and currently being actively developed. Unfortunately, some resources are not fully translated to English yet, but nevertheless the portal contains various useful databases. Of those, GlycoPOD is of particular use for wet-lab scientists. GlycoPOD is a collection of lab protocols for the synthesis or analysis of carbohydrates and other glyco-related experiments. The protocols include step-by-step instructions, references, and features to rate the protocols or to post related questions. Another resource with a special focus on glycochemistry is GlycoNavi, which is a database of chemical reactions that involve carbohydrates and of the molecules involved in these reactions. Information about the chemical properties of carbohydrate molecules can also be found in general molecule databases, such as ChEBI (Chemical Entities of Biological Interest, http://www.ebi.ac.uk/chebi/) [43–44] or PubChem (http://pubchem.ncbi.nlm.nih.gov) [45]. These resources provide data that are important for chemists but are often not present in the more biology-focused carbohydrate-specific databases, such as atomic descriptions, charges, chemical synonyms, SMILES (Simplified Molecular Input Line Entry System) and InChi (IUPAC International Chemical Identifier) codes, and 3D structural information in mol2 format. However, no carbohydrate-specific search options are available. This can make it difficult to locate entries in ChEBI or PubChem especially for oligosaccharides. Introducing cross-links between carbohydrate-specific databases and the major chemical databases would not only make it easier to find specific carbohydrates, but also provide a linkage between biological and chemical information.

#### Databases on glycosyltransferases and glycan binding proteins

As an alternative or complement to chemical synthesis it is possible to make use of the enzymes that build or degrade the glycan chains in vivo, the glycosyltransferases or glycoside hydrolases, respectively [46–49]. To plan such experiments, however, detailed knowledge of the substrate-specificity of these enzymes is required. The same applies to glycan-binding proteins, which can be promising targets for the synthesis of glycomimetics. To some extent, knowledge of such proteins can of course be found in classical protein or enzyme databases, such as UniProt (http://www.uniprot.org) [50] or BRENDA (http://www.brenda-enzymes.info) [51]. However, these databases do not offer any glyco-specific search options. Therefore, it can be difficult to find the respective data in these general databases. There are various resources available that specifically deal with glyco-enzymes or glycan-binding proteins (Table 2). These are often much better suited as starting points for searching than the more general protein databases, not only because of the more narrow focus, but also because most of the glyco-specific protein databases contain links to corresponding entries in the more general databases, but usually not vice versa.

**Table 2:** Glycosyltransferase/glycan-binding protein databases.

Name, Ref	Main content, comments	URL	Status^a^

**Glycosyltransferase (GT) databases**			

CAZy (Carbohydrate Active enZYmes) [52]	Glyco-enzymes clustered into families by sequence comparison	http://www.cazy.org	D/M
CAZyPedia	Wikipedia-like description of GT and GH families	http://www.cazypedia.org	D/M
CFG GT DB	Enzymes for biosynthesis of mammalian glycans	http://www.functionalglycomics.org/glycomics/molecule/jsp/glycoEnzyme/geMolecule.jsp	S
GlycoGeneDB (JCGGDB)	Glyco-enzymes: genes, substrates, gene expression	http://riodb.ibase.aist.go.jp/rcmg/ggdb/	D/M
GPI Biosynthesis report [53]	Enzymes involved in biosynthesis of glycosyl phosphatidyl inositol (GPI) anchors	http://mendel.imp.ac.at/SEQUENCES/gpi-biosynthesis/	S
KEGG Pathway	Biosynthesis pathways, enzyme entries with sequence and notation data and links to other resources	http://www.genome.jp/kegg/pathway.html	D/M
KEGG Orthology	General data on enzymes and catalyzed reactions, links to specific proteins	http://www.genome.jp/kegg/ko.html	D/M

**Glycan binding proteins (GBP) databases**			

CFG Glycan Binding Proteins	Includes information on recognized glycan epitopes and on related diseases	http://www.functionalglycomics.org/glycomics/molecule/jsp/gbpMolecule-home.jsp	S
Genomics Resource for Animal Lectins	Description of animal lectin families	http://www.imperial.ac.uk/research/animallectins/	S
GlyAffinity	Collection of glycan array data from several resources	http://worm.mpi-cbg.de/affinity/	S
GlycoEpitopeDB [54]	Antibodies that bind to carbohydrates, glyco-epitopes recognized by the antibodies	http://www.glyco.is.ritsumei.ac.jp/epitope/	D/M
KEGG BRITE: Glycan Binding Proteins	Protein classification, links to other KEGG resources and to external databases	http://www.genome.jp/kegg-bin/get_htext?ko04091.keg	D/M
Lectin Frontier Database (JCGGDB)	Includes glycan array data	http://riodb.ibase.aist.go.jp/rcmg/glycodb/LectinSearch	D/M
LECTINES	Collection of lectin 3D structures from the PDB	http://www.cermav.cnrs.fr/lectines/	D/M
PACDB (JCGGDB Pathogen Adherence to Carbohydrate DB)	Pathogen adherence molecule, host glycan/glycoprotein ligand, bibliography	http://jcggdb.jp/search/PACDB.cgi	D/M

^a^D/M: Database is further developed and/or maintained; S: Development/maintenance is stopped (or, in the case of CFG resources, will be stopped soon because funding will discontinue).

A major resource for glyco-enzymes (glycosyltransferases, glycoside hydrolases, polysaccharide lyases and carbohydrate esterases) as well as proteins that feature carbohydrate-binding modules is CAZy (Carbohydrate Active Enzymes). This database classifies proteins by sequence comparison and clusters them into families by using well-established bioinformatics tools such as BLAST [55] or HMMER [56]. In this way, approximately 1–3% of the proteins encoded by a typical genome are categorized as glyconzymes [52,57]. For each CAZy family, the corresponding proteins are listed (and can be filtered by subcategories, such as taxonomic kingdoms or entries with existing 3D structural information) together with links to corresponding entries in NCBI GenBank, UniProt [50] or PDB [40]. However, little information is provided about enzyme specificity, kinetics, or catalytic residues, which is crucial information if the enzymes are to be used in carbohydrate synthesis experiments. Such information can be obtained together with literature references from CAZy’s sister resource CAZypedia, a wiki on glyco-enzymes.

Glyco-enzyme data are also found in KEGG Pathway and KEGG Orthology. These resources are not glyco-specific, but metabolic pathways are classified in a hierarchical system, which makes it easy to locate the glyco-related data, but also to learn about the relations between carbohydrate metabolism and other metabolic pathways. KEGG resources cover a diverse range of species of all kingdoms. In contrast, CFG GT database focuses on mammalian glycosyltransferases, and GT information in ECODAB [23] is limited to *E. coli* enzymes. GlycoGeneDB as part of the JCGGDB portal also holds data on glyco-enzymes, including information on substrate specificity, which is important when the enzymes are to be used to synthesize glycan structures in the lab.

Information on glycan-binding proteins (GBPs) or lectins is stored in various databases such as CFG GBP DB, GlycoEpitopeDB [54], the Glycan Binding Proteins section of KEGG BRITE, Lectin Frontier Database, and GlyAffinity. KEGG BRITE mainly links to other resources within and outside the KEGG portal, providing protein sequences, classifications, and information regarding related diseases. GlycoEpitopeDB provides information on antibodies that recognize specific carbohydrate epitopes and glycoproteins or glycolipids that are known to carry the epitopes.

A frequently used technique to study the epitopes, to which a GBP binds, are glycan arrays [4]. CFG Glycan Binding Proteins DB and Lectin Frontier Database store data of glycan array experiments and thus also provide information on the glycan specificity of GBPs. Glycan array data from these two resources and from other research groups are collected and available via a common interface in GlyAffinity. PACDB (Pathogen Adherence Carbohydrate Database) lists glycan-binding proteins that are involved in the adherence of pathogens to the host. The data are extracted from the literature and can be accessed by pathogen names or by related diseases.

### Integration of carbohydrate databases

It is obvious that a lot of knowledge on carbohydrates is stored in the databases, but also that this knowledge is widely spread over the resources. In contrast to genomics or proteomics databases, hardly any exchange of data is carried out between glycan databases. However, some attempts have been made to cross-reference corresponding entries or to allow cross-database searches. As already mentioned above, the individual databases of JCGGDB are cross-linked with each other, as well as the different KEGG resources. Links between these two initiatives also exist.

Cross-references have also been established between distinct resources. BCSDB and Glycosciences.DB, for example, allow cross-database searches, in which users can simultaneously query both resources [58]. Furthermore, there are links available between corresponding entries of the CFG Glycan Structure Database and Glycosciences.DB, but these links are not updated any more, i.e., recently added entries are not covered. Despite these first attempts, it is still rather cumbersome to search for information on a specific glycan structure, as most resources have not only developed individual interfaces, to which the user has to adapt, but they also use individual ways to encode the carbohydrate structures. CarbBank, for example, used a two-dimensional notation that is similar to IUPAC extended notation [59]. This notation is relatively easy for the human user to survey, but is difficult to handle computationally. Therefore, most databases have developed more clearly defined notations to store carbohydrate structures, such as the LINUCS notation [60] of Glycosciences.de, the LinearCode^®^ used within the CFG databases, GlycoCT [61] in EUROCarbDB, or KCF [62] in KEGG GLYCAN. The usage of individual notations is one of the main reasons that hamper the integration of carbohydrate databases. Conversion of one notation to another is often difficult because they do not only differ in the way in which the linkages are encoded and the branching is handled, but also in the denotation of residue names. Dictionaries of frequently occurring monosaccharides can be created manually, but unusual residues, as can be found in bacterial or synthetic glycans, are difficult to handle in this way. This issue is tackled by MonosaccharideDB, which provides routines to automatically parse and encode carbohydrate residues in various notations.

GlycomeDB [28–30] aims to overcome the problem of poor integration of carbohydrate databases by collecting carbohydrate structures and taxonomy data from other databases, namely BCSDB, CarbBank, CFG, EUROCarbDB, GlycoBase (Lille), Glycosciences.DB, and KEGG GLYCAN. Carbohydrate-containing PDB entries are also included by extracting this information from Glycosciences.DB. The glycan structures are translated to a common notation (GlycoCT) by using manually curated dictionaries and MonosaccharideDB routines. Glycans are also stored in GLYDE-II encoding [63–64], which was agreed on as a general carbohydrate data-exchange format [9]. All structures in GlycomeDB can be accessed via a common interface, which allows searches by (sub-)structure, similarity, maximum common substructure, and species. Individual entries mainly provide links to the original database entries in the resources, from which the structures were obtained. This way, GlycomeDB serves as a search engine that allows users to easily navigate through several databases without having to query all resources individually. However, it does not contain further data beyond structural and taxonomic information. Integrating more data offers the possibility of performing systems biology analyses. Such approaches are served by JCGGDB, as already mentioned above, and the newly founded UniCarbKB project [65]. At the time of writing this article, however, UniCarbKB is still in a very early stage.

### Statistical analyses of carbohydrate databases aid the planning of glycan synthesis

Information extracted from glycan structure databases can be useful for glycochemists not only to find potential synthesis targets but also to plan efficient synthesis approaches by providing lists of building blocks that are minimally necessary to synthesize a large number of glycan structures stored in the databases. An analysis of mammalian carbohydrate structures present in Glycosciences.DB, for example, revealed that this data set contained 3299 oligosaccharides, which are part of *N*-glycans, *O*-glycans or glycolipids from 38 mammalian species. Only ten different monosaccharides were found in this data set [66]. However, different anomeric configurations and some substitutions, such as sulfate groups, were ignored, and no distinction was made between *N*-acetylneuraminic acid (Neu5Ac) and *N*-glycolylneuraminic acid (Neu5Gc). The large number of different oligosaccharides that are formed from this relatively small number of different residues arises from the fact that the monosaccharides can be linked in several ways, which has to be considered when creating a set of building blocks for the chemical synthesis of these glycans. Nevertheless, 25 building blocks are sufficient to synthesize 60% of the mammalian glycans stored in the database, and with 36 building blocks 75% of the glycans can be created chemically [66].

The situation is much more complex where bacterial carbohydrates are concerned. The variety of different monosaccharides as well as of different disaccharide pairs that are present in bacterial glycomes is significantly larger than in the mammalian glycome, featuring many residues that do not occur in mammals, but also exhibiting differentiation between individual classes of bacteria [1–2]. Due to the complexity of residue notation this structural diversity is a challenge for glycoinformatics, but it also offers many possibilities to synthesize carbohydrates or glycomimetics that target specific pathogen proteins. For example, oligosaccharide motifs that are found in surface carbohydrates of pathogens, but not in host organisms or in symbiotes, can serve as templates in vaccine development [67–70], and glycomimetics that block specific enzymes or lectins can be used for therapeutic purposes [71–77].

The Glycan Pathway Prediction (GPP) tool of the RINGS portal [78] can be used to predict glycans that can be obtained with a given glycan structure and a set of enzymes. If knowledge of gene expression is available, e.g., from gene microarray experiments, KEGG Gene Expression to Chemical Structure (GECS) can be used to predict the *N*-glycan chains that can be created by the expressed glyco-enzymes. Further tools that are available for the analysis and conversion of glycan sequence data are summarized in Table 3. The Glycan Fingerprints approach to calculate the degree of diversity in a set of glycan structures is a useful tool to, e.g., evaluate the glycans that are present on a glycan array [79]. GlycanBuilder [80] and DrawRings are used by some databases to enable graphical input of glycan (sub-)structure queries using icons to describe monosaccharides. Atomic pictograms as frequently used by chemists, however, are not supported by these tools.

**Table 3:** Tools to input, convert, or analyze glycan structures.

Name, Ref	Comment	URL

DrawRings	Visual editor of glycan structures	http://rings.t.soka.ac.jp/cgi-bin/tools/DrawRings/drawrings2.pl
GECS (Gene Expression to Chemical Structure) [81]	Prediction of *N*-glycan chains from gene expression data	http://www.genome.jp/tools/gecs/
GlycanBuilder [80]	Visual editor of glycan structures	http://www.glycoworkbench.org/wiki/GlycanBuilder
Glycan Fingerprints [79]	Estimation of the degree of diversity in a set of glycan structures	–
Glycan Miner [82]	Detection of motifs or significant subtrees in a set of glycan structures	http://rings.t.soka.ac.jp/cgi-bin/tools/GlycanMiner/Miner_index.pl
GPP (Glycan Pathway Predictor)	Computes *N*-glycan biosynthesis pathway for a given glycan structure	http://rings.t.soka.ac.jp/cgi-bin/tools/GPP/gpp_index.pl
LiGraph	Builds graphical representations of glycans	http://www.glycosciences.de/tools/LiGraph/
ProfilePSTMM [83]	Generates glycan profiles from glycan structure data	http://rings.t.soka.ac.jp/cgi-bin/tools/ProfilePSTMM/profile-training_index.pl
Sumo (Sugar Motif Search)	Detects frequently occurring motifs in a glycan structure	http://www.glycosciences.de/tools/sumo/

### 3D Structure information for targeted drug design

Knowledge of the 3D structure of the target protein and its ligand is a prerequisite for a targeted design of therapeutic glycomimetics [74]. Protein 3D structures are stored in the Protein Data Bank (PDB, [40]). The PDB offers various options to search for proteins. Finding specific carbohydrate structures within PDB entries, however, can be difficult when using PDB queries only. Instead, glycan databases that provide links to PDB entries such as GlycoconjugateDB or Glycosciences.DB can be used. The LECTINES database lists PDB entries of lectins grouped by lectin families. Unfortunately, carbohydrate moieties in the PDB are of significantly lower quality than the protein parts [26,84–86]. Reasons for this are both the greater complexity of carbohydrates, and the fact that, while numerous validation tools are available for protein structures [87], only a few programs exist to validate carbohydrate 3D structures. The PDB carbohydrate residue check (pdb-care) tool [88] aids researchers with locating errors in carbohydrate 3D structures (3D structure-related tools are summarized in Table 4). Ramachandran-like plots of glycosidic torsions are generated by CARP [89], which compares torsions observed in a given 3D structure with computationally derived conformational maps of GlycoMapsDB [27] or with torsions present in carbohydrates in the PDB provided by glyTorsion [89]. In contrast to protein backbone torsions, unusual glycosidic torsions do not necessarily indicate errors in the 3D structure because the conformation of a carbohydrate ligand in complex with a protein can differ from the preferred conformation in solution [90–91]. Nevertheless, CARP plots can help researchers to find potential problems, as well as indicate unusual binding conformations that have to be taken into account when planning the synthesis of glycomimetics.

**Table 4:** Tools for prediction and analysis of carbohydrate/glycoprotein 3D structures.

Name, Ref	Comment	URL

BALLDock/SLICK [92–93]	Protein–carbohydrate docking	
CARP [89]	Ramachandran plot-like analysis of glycosidic torsions	http://www.glycosciences.de/tools/carp/
CAT	Conformational analysis tool, for analysis of MD trajectories	http://www.md-simulations.de/CAT/
GLYCAM Biomolecules Builder [42]	Generation of glycan models and in silico glycosylation of proteins, preparation of input files for AMBER [94–95]	http://glycam.ccrc.uga.edu/ccrc/pages/3dspt.html
Glycan Reader [96]	Detection of carbohydrates in PDB files, preparation of input files for CHARMM [97]	http://www.charmm-gui.org/input/glycan
glyProt [98]	in silico glycosylation of proteins	http://www.glycosciences.de/modeling/glyprot/
glyTorsion [89]	Statistics of torsion angles of carbohydrate structures in the PDB	http://www.glycosciences.de/tools/glytorsion/
glyVicinity [89]	Amino acids in the spatial vicinity of carbohydrates in the PDB	http://www.glycosciences.de/tools/glyvicinity/
pdb2linucs [85]	Detection of carbohydrates in PDB files	http://www.glycosciences.de/tools/pdb2linucs/
pdb-care [88]	Validation of carbohydrate 3D structure files	http://www.glycosciences.de/tools/pdbcare/
Sweet-II [39]	Prediction of carbohydrate 3D structures	http://www.glycosciences.de/modeling/sweet2/

In many cases, however, no carbohydrate ligands are present in PDB entries of glycan-binding proteins. Glycan chains of glycoproteins are also often missing, or only a fraction of a chain is present in the coordinates. In such cases, tools such as Sweet-2 [39] or GLYCAM Biomolecule Builder [42] can be used to create models of carbohydrate chains. The latter program can also perform in silico glycosylation by adding the glycan chains to a protein 3D structure, and provides input files for the AMBER [94–95] modeling programs using the GLYCAM force field [99]. Glycan 3D structures calculated by Sweet-2 can be linked to a protein with glyProt [98]. When using these tools to create conformational models of carbohydrates or glycoproteins, one should always keep in mind that these are models and do not represent the one and only “correct” conformation. As glycans are rather flexible molecules, they adopt several conformations with different populations. The conformational space of a glycan can be analyzed by molecular dynamics (MD) simulations (see in the following) [100]. For this purpose the models generated by the GLYCAM Biomolecules Builder are convenient, as this tool already provides the input files for AMBER simulations. The list of residues that are available, however, is more limited than in Sweet-2. Sulfated residues, which frequently occur in glycosaminoglycans [101], for example, are only supported by Sweet-2 at the moment. GlycanReader [96] as part of the CHARMM-GUI [102] creates CHARMM [97] input files from PDB files that contain carbohydrates. Various tools to predict the occupancy state of potential glycosylation sites from protein sequence data are available as well (Table 5).

**Table 5:** Prediction and analysis of glycosylation sites.

Name, Ref	Comment	URL

big-PI [103]	GPI anchor modification site prediction	http://mendel.imp.ac.at/sat/gpi/gpi_server.html
CBS prediction servers	Collection of various prediction tools, including NetNGlyc, NetOGlyc [104], NetCGlyc [105], NetGlycate [106], DictyOGlyc [107], YingOYang [108]	http://cbs.dtu.dk/services/
CKSAAP_OGlySite [109]	Prediction of mucin-type O-glycosylation sites	http://bioinformatics.cau.edu.cn/zzd_lab/CKSAAP_OGlySite/
EnsembleGly [110]	Prediction of O-, N-, and C-linked glycosylation sites	http://turing.cs.iastate.edu/EnsembleGly/
glySeq [89]	Statistical analysis of amino acids around glycosylation sites	http://www.glycosciences.de/tools/glyseq/
GPI-SOM [111]	Identification of GPI-anchor signals	http://gpi.unibe.ch
GPP [112]	Prediction of N- and O-glycosylation sites	http://comp.chem.nottingham.ac.uk/glyco/

If a protein–carbohydrate complex is to be modeled, generally available docking tools such as AutoDock [113] can be used to identify the binding position. These tools, however, often do not sufficiently consider the peculiarities of protein–carbohydrate complexes, such as CH–π interactions [13]. Therefore, BALLDock/SLICK has been developed specifically for protein–carbohydrate docking [92–93]. One of the major problems of docking algorithms in general is the identification of the correct conformation among the potential binding modes [100]. Therefore, computational docking approaches are frequently combined with wet-lab experiments, such as saturation transfer difference NMR (STD NMR) or transferred nuclear Overhauser effect (trNOE) spectroscopy [75,114–116], to reliably assign the correct conformation of the ligand on the protein surface. Such combinations of experimental and theoretical approaches are also useful to determine the conformations of natural carbohydrates or their synthetic glycan mimetics [117–119]. Results can be improved by combinations of different modeling approaches, such as docking and MD simulations [101]. To run reliable MD simulations of carbohydrate 3D structures, force fields are necessary that contain parameters for carbohydrates. In the case of glycoproteins, protein–carbohydrate complexes, or glycolipids, the force fields have to cover all types of molecules involved [100,120]. The force fields need to be extensible if not only standard monosaccharides, but also derivatized residues are included in a simulation, which is especially important during the design of glycomimetics [121]. Parameters that affect the simulations of carbohydrates include the treatment of atom charges [122], and solvent model (several models for water are available) [100]. The question of whether to include extra terms for (exo) anomeric effects has also been discussed for a long time [13].

With increasing computational power, MD simulations of larger molecules become feasible; and timescales of simulations increase. One major bottleneck for the scientific use of MD simulations that involve carbohydrates is, therefore, the availability of tools to analyze these simulation trajectories [13]. MD software packages contain analysis tools [94,97,123], but these are tailored for analyzing simulations of proteins. Therefore, tools such as CAT (Conformational Analysis Tools) that serve the needs of glycoscientists are specifically developed [13].

### Glycoinformatics in carbohydrate structure analysis

After synthesis of carbohydrate chains an assessment of the quality of the produced material is necessary to exclude wrong structures among the products. Errors in structures that are meant for use in experiments such as glycan arrays may yield incorrect results and thereby lead to mistaken conclusions. Incorrect products that are used as therapeutics can have severe results. Impurities in heparin, a widely used carbohydrate pharmaceutical, for example, can even be fatal [124–126]. The major methods for quality control comprise MS, NMR, and HPLC, all of which produce large amounts of data that have to be evaluated. Companies that develop the analytical equipment that is necessary for these techniques usually do not focus on the detection or analysis of carbohydrates and their software does not suit the needs of glycoscientists [12]. Nevertheless, various community-developed tools exist that facilitate the carbohydrate-specific interpretation of these data (Table 6).

**Table 6:** Tools to support experimental analysis of glycans.

Name, Ref	Comment	URL

**Mass spectrometry**		

Cartoonist [127–128]	Template based glycan sequencing	–
GlycanMass	Calculates the mass of an oligosaccharide structure	http://web.expasy.org/glycanmass/
GLYCH [129]	De novo sequencing of glycans	–
GlycoFragment [130]	Calculation of theoretical mass fragments of glycans	http://www.glycosciences.de/tools/GlycoFragments/
GlycoMiner [131]	Glycopeptide (*N*-glycan) composition analysis	http://www.chemres.hu/ms/glycominer/
GlycoMod [132]	Prediction of oligosaccharide structures of glycoproteins from mass peaks	http://www.expasy.org/tools/glycomod/
Glyco-Peakfinder [133]	Composition annotation of glycans in MS spectra	http://www.glyco-peakfinder.org
GlycoPep ID [134]	Glycan mass fingerprinting	http://hexose.chem.ku.edu/predictiontable.php
GlycoPeptideSearch [135]	Glycan mass fingerprinting (MS/MS) of *N*-glycopeptides using GlycomeDB glycans	http://edwardslab.bmcb.georgetown.edu/software/GlycoPeptideSearch.html
Glyco-Search-MS [136]	Glycan mass fingerprinting using Glyocsciences.DB glycans	http://www.glycosciences.de/database/start.php?action=form_ms_search
GlycosidIQ [137]	Glycan mass fingerprinting using GlycoSuiteDB glycans	–
GlycoWorkbench [138]	Assists interpretation of MS spectra	http://www.glycoworkbench.org
GlyQuest [139]	Glycopeptide (*N*-glycan) analysis	–
MS^n^ FragLib [140]	Glycan characterization based on an MS^n^ fragment spectral library	–
OSCAR [141]	De novo sequencing of glycans	–
Peptoonist [127]	Identification of *N*-glycopeptides from a series of mass spectra (MS and MS/MS)	–
PMAA (Partially Methylated Alditol Acetate)	GC–MS fragmentation of permethylated monosaccharides	http://www.ccrc.uga.edu/specdb/ms/pmaa/pframe.html
STAT [142]	De novo sequencing of glycans	–
StrOligo [143]	De novo sequencing of glycans	–

**NMR**		

CASPER [144–145]	Simulation of NMR spectra, glycan sequence determination from chemical shifts	http://www.casper.organ.su.se/casper/
CCPN [146]	NMR annotation software	http://www.ccpn.ac.uk
GlyNest [147]	Estimation of NMR chemical shifts	http://www.glycosciences.de/sweetdb/start.php?action=form_shift_estimation
ProspectND	NMR data processing and inspection	http://prospectnd.sourceforge.net/

**HPLC**		

AutoGU [25]	Interpretation of HPLC data	–
GALAXY [148]	Visualization of HPLC 2D maps	http://www.glycoanalysis.info/ENG/index.html

Among the three techniques, the largest choice of tools is available for mass spectrometry. These programs commonly first try and assign residue compositions to measured mass peaks, but use different approaches to determine glycan sequences from compositional data. Some tools such as Cartoonist [127–128] and GlycoMod [132] apply constraints that are created from the knowledge of biosynthetic pathways, thus they are tailored to the analysis of biological samples rather than of chemically synthesized glycans, which do not match the biosynthetic pathways. In contrast, the mass fingerprinting approach as implemented in GlycosidIQ [137], GlypPep ID [134], or Glyco-Search-MS [136] works similarly to algorithms that are frequently used in peptide or protein identification by tools such as Mascot: Mass peaks that are observed in a spectrum are compared to theoretically derived fragment masses that are computed from glycan structures stored in a carbohydrate database. This approach, however, is limited by the content of the database that provides the templates for in silico fragmentation, which means that structures that have not been observed before or that are missing from the database will not be identified this way. This problem also applies to programs that use experimental MS^n^ data of oligosaccharide standards to assign MS^n^ fragments of larger glycans, such as GLYCH [129] or MS^n^ FragLib [140]. In contrast, programs such as STAT [142], StrOligo [143], or OSCAR [141] also allow de novo determination of structures because they interpret MS^n^ data by determining the possible compositions of parent ions according to their masses; subsequently, the masses of possible connected branching topologies are computed to match the experimentally determined data [149]. GlyQuest [139] and GlycoMiner [131] are designed for high-throughput analysis of glycopeptides that carry *N*-glycan chains. Glyco-Peakfinder [133] and GlycoWorkbench [138] cover the complete workflow from recorded experimental data to a fully assigned spectrum or to glycan structure determination [150]. GlycoWorkbench also facilitates upload of primary data into EUROCarbDB [24]. Furthermore, it enables user-defined residues, which is important when chemically synthesized glycans are to be analyzed. Such glycans may contain highly modified monosaccharides, protecting groups, linkers, or other kinds of nonstandard residues, which are neither included in the standard residue sets nor present in the databases used by mass-fingerprinting approaches.

Significantly fewer tools are available to aid the interpretation of HPLC or NMR spectra of carbohydrates. AutoGU [25] and GALAXY [148] assist users to interpret HPLC profiles or to visualize HPLC 2D maps, respectively. ProspectND is designed for the signal processing of multidimensional NMR spectra. CCPN (Collaborative Computing Project for the NMR community) helps users to assign NMR spectra [146,151]. Originally designed for proteins or peptides, CCPN by now also supports carbohydrates. Other tools such as CASPER [144–145,152–154] or GlyNest [147] can be used to predict 1D NMR spectra of carbohydrates and to determine glycan sequences from chemical shifts.

## Conclusion

Due to the challenges that carbohydrates pose, not only with respect to their analysis or synthesis but also in the handling of them computationally, glycoinformatics has been lagging behind other areas of bioinformatics for a long time [6], but has made good progress over the past decade and is catching up with bioinformatics for genomics or proteomics. Despite this relatively quick growth and some promising approaches to cross-reference and standardize the data [9–10,28,58,65], there is still an urgent need for better integration of the various resources [13], many of which can still be regarded as disconnected islands. Furthermore, funding for the maintenance of existing databases is required to keep useful resources up-to-date, rather than only funding new projects. The open-source idea can also help to partly overcome this dilemma. If the data and source codes that have been developed in a project are accessible to other researchers, they can be used in new projects to actually improve the existing status, rather than the wheel having to be reinvented every time by starting from scratch and redeveloping basic concepts and sources.
